# Gonadal transcriptomes reveal sex-biased expression genes associated with sex determination and differentiation in red-tail catfish (*Hemibagrus wyckioides*)

**DOI:** 10.1186/s12864-023-09264-x

**Published:** 2023-04-06

**Authors:** Wen-Yu Wei, Yi Gong, Xin-Fen Guo, Min Liu, Yu-Lin Zhou, Zhi Li, Li Zhou, Zhong-Wei Wang, Jian-Fang Gui

**Affiliations:** 1 College of Fisheries and Life Science, Dalian Ocean University, Dalian, 116023 China; 2 State Key Laboratory of Freshwater Ecology and Biotechnology, Hubei Hongshan Laboratory, The Innovation Academy of Seed Design, Institute of Hydrobiology, Chinese Academy of Sciences, Wuhan, 430072 China; 3 University of Chinese Academy of Sciences, Beijing, 100049 China

**Keywords:** *Hemibagrus wyckioides*, RNA-Seq, Gonadal transcriptome, Sex differentiation, Sex determination

## Abstract

**Background:**

Red-tail catfish (*Hemibagrus wyckioides*) is an important commercially farmed catfish in southern China. Males of red-tail catfish grow faster than females, suggesting that all-male catfish will produce more significant economic benefits in aquaculture practice. However, little research has been reported on sex determination and gonadal development in red-tail catfish.

**Results:**

In this study, we performed the first transcriptomic analysis of male and female gonads at four developmental stages at 10, 18, 30, and 48 days post hatching (dph) using RNA-seq technology. A total of 23,588 genes were screened in 24 sequenced samples, of which 28, 213, 636, and 1381 differentially expressed genes (DEGs) were detected at four developmental stages, respectively. Seven candidate genes of sex determination and differentiation were further identified. Real-time quantitative PCR (RT-qPCR) further confirmed that anti-Mullerian hormone (*amh*), growth differentiation factor 6a (*gdf6a*), testis-specific gene antigen 10 (*tsga10*), and cytochrome P450 family 17 subfamily A (*cyp17a*) were highly expressed mainly in the male, while cytochrome P450 family 19 subfamily A polypeptide 1b (*cyp19a1b*), forkhead box L2 (*foxl2*), and hydroxysteroid 17-beta dehydrogenase 1 (*hsd17b1*) were highly expressed in the female. The KEGG pathway enrichment data showed that these identified DEGs were mainly involved in steroid hormone biosynthesis and TGF-β signaling pathways.

**Conclusions:**

Based on RNA-seq data of gonads at the early developmental stages, seven DEGs shared by the four developmental stages were identified, among which *amh* and *gdf6a* may be the male-biased expression genes, while *foxl2*, *cyp19a1b* and *hsd17b1* may be the female-biased expression genes in red-tail catfish. Our study will provide crucial genetic information for the research on sex control in red-tail catfish, as well as for exploring the evolutionary processes of sex determination mechanisms in fish.

**Supplementary Information:**

The online version contains supplementary material available at 10.1186/s12864-023-09264-x.

## Background

Red-tail catfish (*Hemibagrus wyckioides*), a freshwater species of the family *Hemibagrus*, Bagridae, Siluriformes, is mainly distributed in multiple Southeast Asian countries and the Lancang River of the Yunnan Province, China [1, 2]. Owing to its high protein content, excellent production, strong disease resistance, and absence of intermuscular bones, red-tail catfish has shown a sustained increase in its culture in southern China and has become a momentous economic aquaculture species in China in recent years [3, 4]. Like other catfish species, such as yellow catfish (*Pelteobagrus fulvidraco*) [5, 6], channel catfish (*Ictalurus punctatus*) [7], Lanzhou catfish (*Silurus lanzhouensis*) [8], and Ussuri catfish (*Pseudobagrus ussuriensis*) [9, 10], red-tail catfish shows sexual dimorphism in growth and sex-specific markers has been identified for gender identification [11]. Sexual dimorphism in growth refers to the difference between males and females of the same species, which is very common in fish, such as Nile tilapia (*Oreochromis niloticus*) [12], half-smooth tongue sole (*Cynoglossus semilaevis*) [13], and blotched snakehead (*Channa maculate*) [14]. In aquaculture practice, it is of great economic significance to breed monosexual fish populations based on the regulation mechanism of sex determination and differentiation of fish [15–18]. Therefore, the identification of sex-specific markers or sex-related genes is necessary to reveal the mechanism of sex determination and differentiation in addition to the identification of physiological sex [19, 20].

Fish exhibit all known forms of vertebrate sex determination to adapt to the variable habitats own to its extreme diversity, that is, fish sex determination patterns can be classified as genotypic sex determination (GSD), environmental sex determination (ESD), and genotypic sex determination with environmental effect (GSD + ESD) [19–23]. The expression of sex determination genes regulates the signal pathways of sex determination and sex differentiation, inducing the development of primordial gonads into ovaries or testes [24]. Therefore, whether genes involved in sex determination are conserved throughout evolution has raised great research interest. Several genes have been confirmed as master genes of sex determination in some fish species, such as *cyp19a1a* in the Nile tilapia (*Oreochromis niloticus*) [25], *dmy* and *gsdfY* gene in the medaka fish [26, 27], *amhr2* in fugu (*Takifugu rubripes*) [28], *sdy* in rainbow trout (*Oncorhynchus mykiss*) [29], *pfpdz1* in yellow catfish (*Pelteobagrus fulvidraco*) [30], and *bcar1* in channel catfish (*Ictalurus punctatus*) [31]. These results indicate that genes involved in sex determination and differentiation in fish vary significantly among genus, which has brought great difficulties to the deep revealing of the mechanism of fish sex determination and differentiation. Moreover, the sex chromosomes are generally poorly differentiated in Siluriformes [32], which makes it more difficult to screen the sex determining genes of red-tail catfish. However, no genes related to sex determination and differentiation have yet been identified in red-tail catfish, suggesting that more or novel sex-related genes should be identified to adequately explain the complex mechanism of sex determination.

Transcriptome sequencing is a cost-effective and time-effective method to screen sex determination-related genes and other causal genes [33–35]. Identification of pathways involved in gonadal development could further illuminate the gene regulatory network controlling sex determination and subsequent maintenance of phenotypic sex [36, 37]. Sex-biased genes are expressed either in one sex or at significantly different levels between two sexes and give rise to different phenotypic sexes, which has provided a mechanism for organisms to produce different adaptive phenotypes on the same genetic background [38, 39]. Therefore, in this study, we performed RNA-seq using testes and ovaries at undifferentiated and differentiated four stages in red-tail catfish. Furthermore, the expression patterns of sex-biased DEGs shared by the four developmental stages were analyzed to investigate the mechanism of sex determination and differentiation, which will help us to better reveal the evolution of sex chromosomes and the mechanism of sex determination in higher vertebrates.

## Results

### Sample collection according to the gonadal differentiation time

**Fig. 1:**
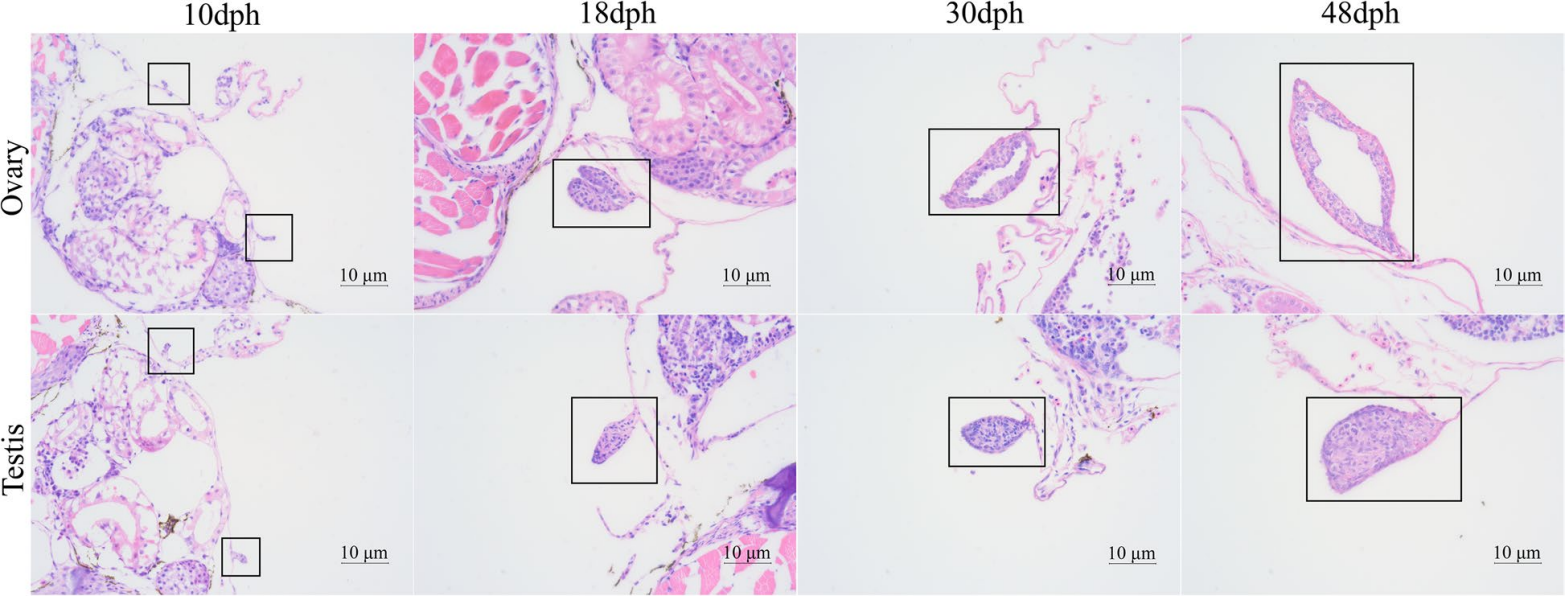
Histological analysis of gonads at four developmental stages. The gonads are highlighted in the black box

The time of gonadal differentiation was determined by histological analysis and it showed that there was no significant difference between female and male gonads before 10dph. At 18dph, female gonads gradually began to form ovarian cavities, but cavity structures did not appear in male gonads. From 30 to 48dph, the ovarian cavity gradually closed in the female gonads, whereas the male gonads still never developed a cavity structure (Fig. 1). It indicated that the gonads of two sexes began to show significantly morphological differences at 18dph or so, suggesting that the initial time of gonadal differentiation is around 18dph in red-tail catfish. Therefore, we sampled testis and ovary tissues at four stages before and after gonadal differentiation, namely, before differentiation (10dph), the initial time of gonadal differentiation (18dph), ovarian cavity fully formed (30dph), after differentiation (48dph). Therefore, a total of 24 cDNA libraries derived from male and female gonads at 10, 18, 30, and 48dph were constructed for transcriptome sequencing.

### Summary statistics of RNA-seq data

The raw data of RNA-seq was deposited in the NCBI database Sequence Read Archive (PRJNA898908). A total of 1,959.69 M raw sequencing reads were generated with 48.49% GC content and Q30 bases distributed between 91.77 and 94.26%. After removing ambiguous nucleotides, 1922.16 M clean reads totaling 284.16 G bases were obtained for the following analysis. Then all the clean reads of each sample were mapped to the reference genome of red-tail catfish with an average total mapping ratio of 94.12 ± 1.23%, ranging from 91.69 to 95.77% (Table 1). In total, 23,588 genes were screened from the 24 sequencing samples for the following DEGs identification.

**Table 1:** Summary statistics of sequencing data

Sample	Raw Reads/M	Clean Reads/M	Clean Bases/G	Q30/%	Total Mapping Ratio/%	Uniquely Mapping Ratio/%
M10dph1	79.53	78.05	11.54	93.38	92.21	87.41
M10dph2	84.30	82.27	12.16	92.88	91.69	87.95
M10dph3	85.47	83.80	12.39	93.14	92.35	87.97
M18dph1	81.29	79.43	11.73	93.19	94.68	90.76
M18dph2	83.19	81.27	12.00	93.35	94.32	90.47
M18dph3	79.12	77.35	11.42	93.35	94.20	90.90
M30dph1	75.89	76.95	11.38	93.72	94.83	91.20
M30dph2	85.07	83.51	12.33	92.38	94.11	90.32
M30dph3	83.62	82.05	12.14	93.98	93.86	89.02
M48dph1	79.09	77.64	11.49	93.82	95.27	91.76
M48dph2	81.44	79.90	11.83	94.21	95.30	91.69
M48dph3	83.62	82.13	12.17	94.26	95.77	92.12
F10dph1	79.17	77.65	11.47	91.77	92.40	87.94
F10dph2	79.38	77.91	11.52	93.22	92.42	86.54
F10dph3	80.63	79.05	11.69	93.48	92.13	88.36
F18dph1	82.47	80.83	11.96	93.41	94.19	90.61
F18dph2	81.96	80.49	11.91	93.81	94.57	91.23
F18dph3	80.28	78.51	11.59	92.62	94.63	91.10
F30dph1	78.69	76.63	11.31	92.83	94.69	91.24
F30dph2	82.47	80.65	11.90	93.37	94.78	91.32
F30dph3	85.27	83.26	12.29	93.31	94.49	90.84
F48dph1	79.87	78.32	11.59	93.75	95.29	91.75
F48dph2	82.44	80.91	11.98	93.89	95.34	91.41
F48dph3	85.43	83.60	12.37	93.88	95.37	91.86

M_dph: male gonad samples at _ days post hatching; F_dph: female gonad samples at _ days post hatching. The numbers “1”, “2” and “3″ after M_dph” and “F_dph” represent three biological replicates. The “Uniquely Mapping ratio” is the statistic of the number of sequenced reads that mapped to only one position on the reference genome

### Identification and functional annotation of DEGs

**Fig. 2:**
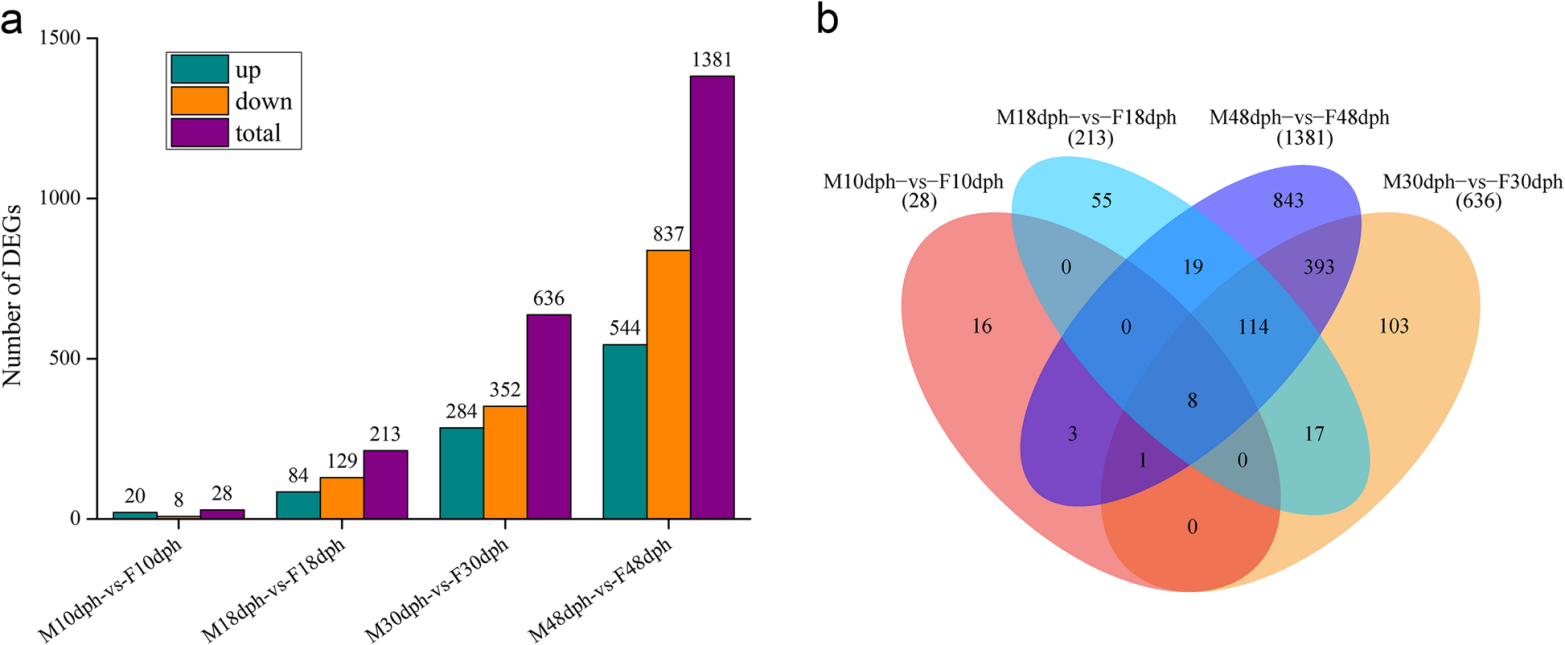
The number of DEGs at four developmental stages. Histogram (**a**) and Venn diagram(**b**)

At four developmental stages, 28, 213, 636, and 1381 DEGs were obtained, respectively (Fig. 2). The number of DEGs continued to increase with gonadal development. In M10dph-vs-F10dph, there were a total of 28 DEGs, of which 20 were up-regulated and 8 were down-regulated. In M18dph-vs-F18dph, there were 213 DEGs in total, of which 84 were up-regulated and 129 were down-regulated. In M30dph-vs-F30dph, there was a total of 636 DEGs, of which 284 were up-regulated and 352 were down-regulated. In M48dph-vs-F48dph, there was a total of 1381 DEGs, of which 544 were up-regulated and 837 were down-regulated. The list of DEGs at four developmental stages was shown in Tables S1-S4.

**Fig. 3:**
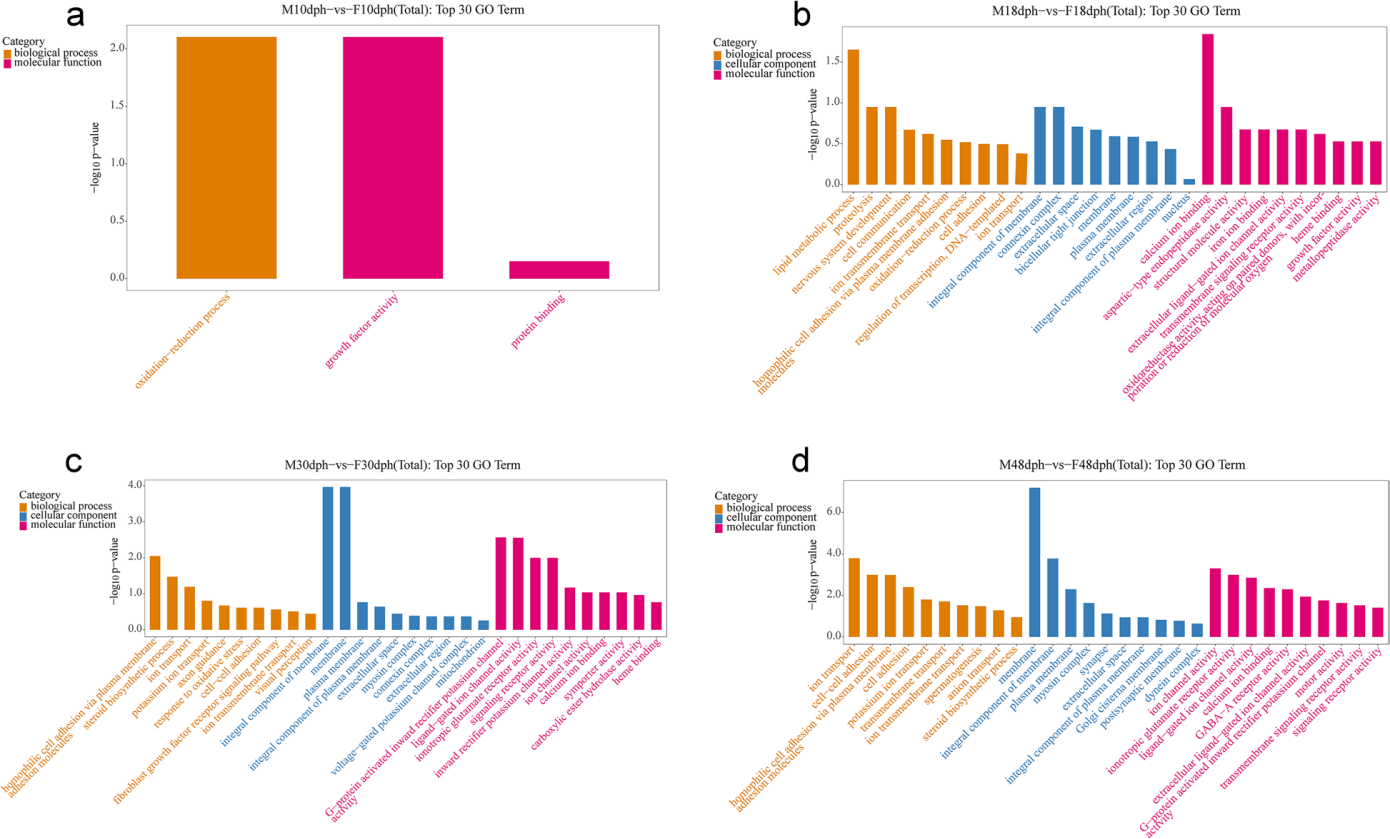
Histogram of top 30 GO terms enriched by DEGs at four developmental stages. M10dph-vs-F10dph (**a**), M18dph-vs-F18dph (**b**), M30dph-vs-F30dph (**c**), and M48dph-vs-F48dph (**d**). The x-axis shows the GO terms and the y-axis indicates negative log of the *p* value of significance. M_dph: male gonad samples at _ days post hatching; F_dph: female gonad samples at _ days post hatching

The GO annotations of the DEGs were classified as molecular function, cellular component and biological process. The DEGs identified from four stages were enriched to 37, 195, 326, and 502 GO terms, respectively. The top 30 enriched GO terms in each period were illustrated in Fig. 3.

**Fig. 4:**
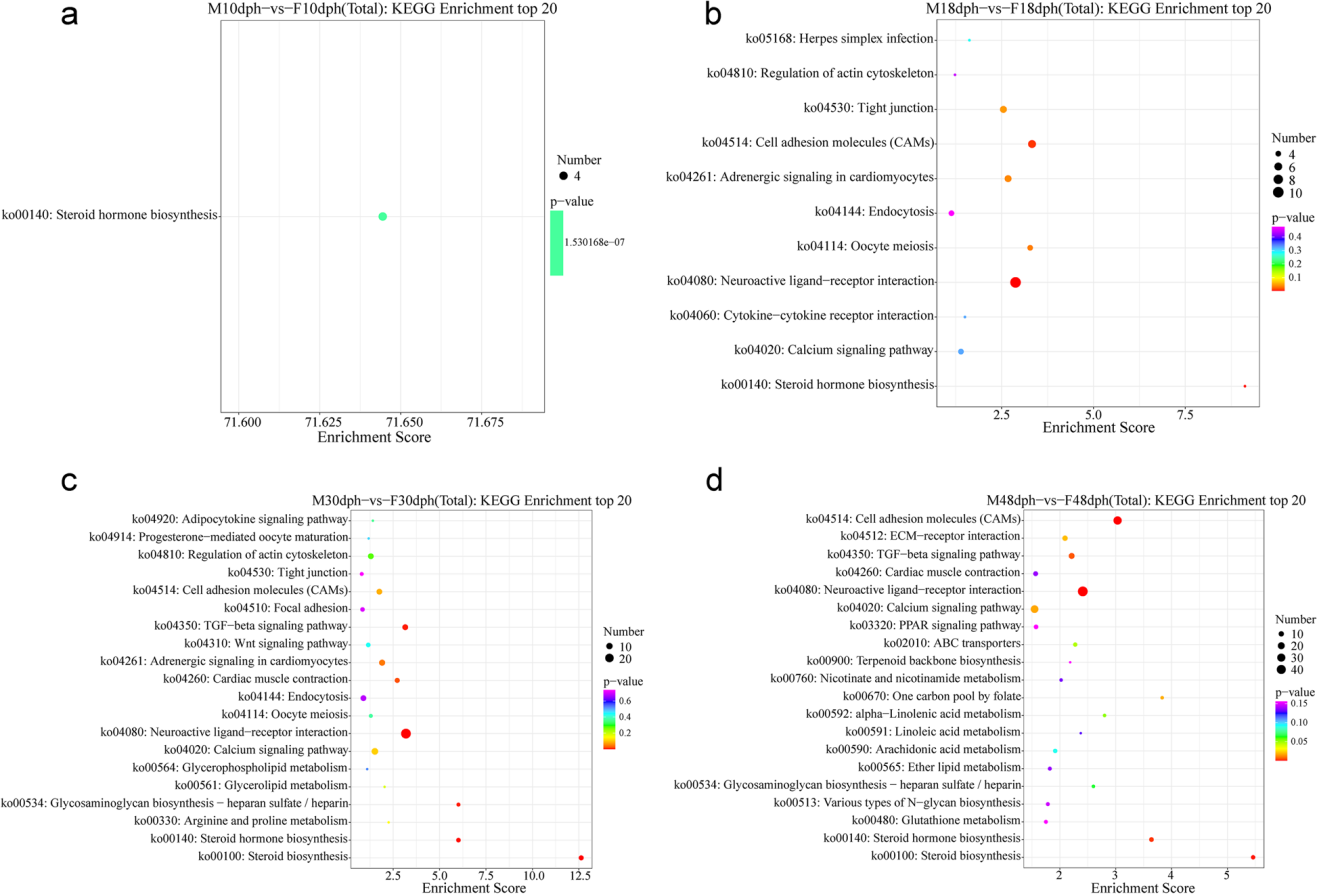
The top 20 KEGG pathways enriched by DEGs at four developmental stages. M10dph-vs-F10dph (**a**), M18dph-vs-F18dph (**b**), M30dph-vs-F30dph (**c**), and M48dph-vs-F48dph (**d**). The x-axis indicates the enrichment score of each pathway and the y-axis shows the pathway. The color and size of dots indicate the *p*-value and number of differentially expressed genes (N ≥ 3) assigned to the corresponding pathway, respectively

The DEGs identified from M10dph-vs-F10dph, M18dph-vs-F18dph, M30dph-vs-F30dph, and M48dph-vs-F48dph were further annotated to 13, 51, 92, and 116 KEGG pathways, respectively. As shown in the top 20 KEGG enrichments, only steroid hormone biosynthesis KEGG pathway was enriched at 10dph (Fig. 4a). The top 3 of 11 significantly enriched pathways were neuroactive ligand-receptor interaction, steroid hormone biosynthesis and cell adhesion molecules (CAMs) at 18dph (Fig. 4b). The top 5 significantly enriched pathways for the DEGs were neuroactive ligand-receptor interaction, steroid biosynthesis, steroid hormone biosynthesis, TGF-β signaling pathway and glycosaminoglycan biosynthesis-heparan sulfate/heparin at 30dph (Fig. 4c). The top 5 significantly enriched pathways for the DEGs at 48dph were essentially identical to those at 18dph except that cell adhesion molecules (CAMs) replaced glycosaminoglycan biosynthesis-heparan sulfate/heparin (Fig. 4d). Among the top 20 KEGG pathways at four developmental stages, steroid hormone biosynthesis pathway was commonly enriched. The DEGs at four developmental stages involved in the steroid hormone biosynthesis pathway were shown in Figs. S1, S2, S3 and S4. In addition, well-known sex-related pathways, such as TGF-β signaling pathway besides steroid hormone biosynthesis, were also identified.

### Identification of sex-biased expression genes

In order to identify male- and female-biased expression genes, the above screened DEGs at four developmental stages were further compared and the DEGs shared by them were considered as sex-biased expression genes. For example, *amh* and *tsga10* were up-regulated at four developmental stages, whereas *cyp19a1b*, *foxl2*, and *hsd17b1* were down-regulated at four developmental stages, suggesting that these genes may be sex-biased expression genes and involved in gonadal differentiation.

To reveal main biological functions and identify biological pathways of the eight shared DEGs of four developmental stages, GO annotation and KEEG enrichment analysis were summarized in Table 2. As *mucin-22-like* was not annotated to the exact sex-related gene, no further analysis of this gene was performed. These seven genes were annotated to eighteen GO terms, such as gonad development, estrogen biosynthetic process, estradiol 17-beta-dehydrogenase activity and spermatogenesis. These genes were enriched in three KEGG pathways, including steroid hormone biosynthesis, TGF-β signaling pathway and cytokine-cytokine receptor interaction.

**Table 2:** GO annotation and KEGG enrichment of eight shared DEGs of four developmental stages

DEGs	GO terms	KEGG pathways
*amh*	a. gonad development b. growth factor activity	a. TGF-β signaling pathway b. cytokine-cytokine receptor interaction
*gdf6a*	a. growth factor activity	a. TGF-β signaling pathway
*cyp17a*	a. heme binding b. iron ion binding c. sex differentiation d. steroid biosynthetic process e. oxidation–reduction process f. steroid 17-α-monooxygenase activity g. 17-α-hydroxyprogesterone aldolase activity h. oxidoreductase activity, acting on paired donors, with incorporation or reduction of molecular oxygen	a. steroid hormone biosynthesis
*cyp19a1b*	a. heme binding b. iron ion binding c. oxidation–reduction process d. oxidoreductase activity, acting on paired donors, with incorporation or reduction of molecular oxygen	a. steroid hormone biosynthesis
*hsd17b1*	a. estrogen biosynthetic process b. estradiol 17-β-dehydrogenase activity c. cytoplasm d. oxidation–reduction process e. oxidoreductase activity	a. steroid hormone biosynthesis
*foxl2*	a. sequence-specific DNA binding b. DNA-binding transcription factor activity c. regulation of transcription, DNA-templated	/
*tsga10*	a. spermatogenesis	/
*mucin-22-like*	/	/

### Verification of the expression patterns of sex-biased genes by RT-qPCR

**Fig. 5:**
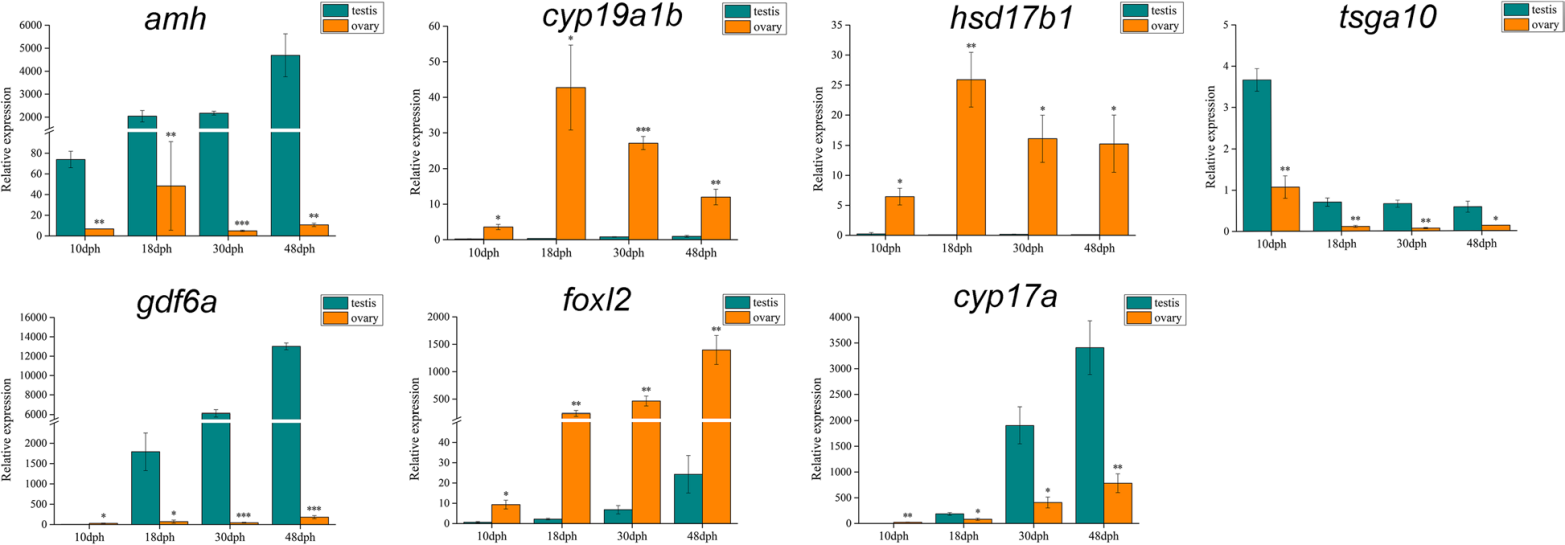
Validation of sex-biased expression of seven DEGs by RT-qPCR. The y-axis indicates relative expression level. Error bars mean standard error. Asterisks represent the level of significant difference between sexes, * (*p* < 0.05), ** (*p* < 0.01), *** (*p* < 0.001). The real *p*-values were shown in Table S5

In total, seven candidate sex-biased expression genes, namely *amh*, *gdf6a*, *tsga10*, *cyp19a1b*, *foxl2*, *hsd17b1,* and *cyp17a* were identified from the DEGs for RT-qPCR to verify the expression patterns in red-tail catfish. The expression levels of the selected seven genes were significantly different between two sexes at each developmental stage. Among these, *amh* and *tsga10* were up-regulated at four developmental stages. The *cyp19a1b*, *foxl2*, and *hsd17b1* were down-regulated at four developmental stages. The expressions of *gdf6a* and *cyp17a* were down-regulated in M10dph-vs-F10dph, but up-regulated in the other three developmental stages (Fig. 5). The gene expression patterns revealed by RT-qPCR results were consistent with the RNA-sequencing data, which indicated that the transcriptome data were reliable and useful for differential expression analysis.

## Discussion

The red-tail catfish shows the characteristics of sex dimorphism in growth, and males show obvious growth advantages. Although sex-specific markers have been developed for genotypic sex identification [11], less research has been focused on the sex determination and differentiation in red-tail catfish. Studies on the mechanism of sex determination provide paramount theoretical value for promoting the development of sex-control breeding. The identification of DEGs at different developmental stages by the gonadal transcriptome is an important way to investigate the molecular differences that regulate sex determination and sexual dimorphism [40]. A total of 28, 213, 636 and 1381 DEGs were identified between two sexes at the four developmental stages through the gonadal transcriptome of the red-tail catfish. There was an increase in the number of DEGs from 10 to 48dph, suggesting that there were more sex-related genes in the later stages of gonadal development compared to the early stages. This trend was similar to that revealed in the closely related fish, such as yellow catfish (*Pelteobagrus fulvidraco*) and channel catfish (*Ictalurus punctatus*) [40, 41].

As genes differentially expressed between two sexes at different developmental stages were involved in gonadal differentiation and development, these sex-related genes can be used to further identify sex determination and differentiation genes [40]. In red-tail catfish, seven candidate genes (*amh*, *gdf6a*, *tsga10*, *cyp17a*, *foxl2*, *cyp19a1b* and *hsd17b1*) were obtained by taking intersections of DEGs at four developmental stages. Two genes (*cyp17a* and *cyp19a1b*) shared during gonadal development in yellow catfish (*Pelteobagrus fulvidraco*) and two genes (*amh* and *foxl2*) at someone stage in half-smooth tongue sole (*Cynoglossus semilaevis*) were the same with those in red-tail catfish [40, 42]. Besides, the expression of these seven genes also showed different patterns before and after gonadal differentiation (18dph). For example, the expression of *hsd17b1* in the female was higher after gonadal differentiation (48dph) than before gonadal differentiation (10dph), and the expression level was the highest at the initial time of gonadal differentiation (18dph). However, the expression of *gdf6a* in the male was higher after gonadal differentiation (48dph) than before gonadal differentiation (10dph), and its expression level increased with gonadal differentiation and development (from 10 to 48dph).

Most of the identified sex-determining genes and candidate genes are from the TGF-β signaling pathway [43]. In this study, two genes belonging to the TGF-β signaling pathway, *amh and gdf6a* were identified. In zebrafish, *amh* mutants showed a female-biased sex ratio, and the proliferation and differentiation of male germ cells were disordered [44]. It was revealed that overexpression of *amh* in undifferentiated orange grouper (*Epinephelus coioides*) induced testis development [45]. In Japanese eel (*Anguilla japonica*), *amh* mainly expressed in the testis, and the expression level increased significantly with the differentiation of the testis [46]. Male-biased expression of *amh* was associated with the differentiation of the male gonads in yellow catfish (*Pelteobagrus fulvidraco*) [30]. In red-tail catfish, *amh* showed significantly different expression at four developmental stages. Furthermore, as in the southern catfish (*Silurus meridionalis*) [47], *amh* showed significantly higher expression in the male than in the female in red-tail catfish. As for *gdf6a*, most of studies have shown that it is related to the photoreceptor phylogeny of zebrafish (*Danio rerio*) [48–50]. It has been also reported that *gdf6* on the Y chromosome is a master sex-determining gene in the turquoise killifish (*Nothobranchius furzeri*) [51]. In red-tail catfish, the expression of *gdf6a* was down-regulated at the undifferentiated stage (10dph), but up-regulated at the initial time of gonadal differentiation stage (18dph). In conclusion, *gdf6a* and *amh* showed different expression patterns at the undifferentiated stage (10dph), but showed similar expression patterns after gonadal differentiation (18dph) in red-tail catfish. Furthermore, both *amh* and *gdf6a* belong to the TGF-β signaling pathway genes [52]. These results suggest that *amh* may be involved in the molecular sex differentiation, however, *gdf6a* may play a main role in the morphological sex differentiation in red-tail catfish.

Besides TGF-β signaling pathway genes, DM domain containing genes and Sox family genes are also the main source genes of sex-determining genes or candidate genes [19, 43, 53], such as *dmrt1* (a DM domain containing gene) in medaka fish (*Oryzias latipes*) and half-smooth tongue sole (*Cynoglossus semilaevis*) [26, 54, 55], sox3 (a sox family gene) in Indian ricefish (*Oryzias dancena*) [56], and *sox9* (a sox family gene) in medaka fish (*Oryzias latipes*) and orange-spotted grouper (*Epinephelus coioides*) [57–59]. However, these genes were not differentially expressed in the critical period of gonadal differentiation in red-tail catfish. For example, *dmrt1* was not differentially expressed between two sexes at 10dph and 18dph. Although the expression of *dmrt1* were up-regulated at 30dph and 48dph, its expression was kept at a very low level. Similarly, *sox9* was not differentially expressed at a low level at the four stages. Therefore, it is inferred that *dmrt1* and *sox9* may not be sex-determining genes, but play a role in the maintenance of gonadal development in red-tail catfish.

Genes involved in steroid hormone biosynthesis also were key physiological factors in regulating sex differentiation in fish [60]. Fortunately, *cyp17a*, *cyp19a1b*, and *hsd17b1*, belonging to the steroid hormone biosynthesis pathway, were screened from the DEGs in red-tail catfish. Cyp17a controls the synthesis of 17β-estradiol, and *cyp17a* (-/-) XX leads to male sex reversal in Nile tilapia (*Oreochromis niloticus*) [61]. The expression of *cyp17a* in red-tail catfish was similar to that in rice field eels (*Monopterus albus*), but different from that in Nile tilapia (*Oreochromis niloticus*) and fathead minnows (*Pimephales promelas*) [62–64], suggesting that the role for *cyp17a* during gonadal differentiation and development may vary with species or developmental stage. Cyp19 converts androgens to estrogens, and the expression of this gene determines the ratio of androgens to estrogens, which is vital for sex differentiation in most vertebrates [65]. During gonadal development and sex differentiation, treatment with the CYP19 inhibitor fadrozole resulted in gonad differentiation into testes in all individuals of zebrafish (*Danio rerio*) and masculinization in 97.1% of common carp (*Cyprinus carpio*), respectively [66, 67]. In the process of sex differentiation, *cyp19a1* was highly expressed in the female gonad or specifically expressed in the early differentiated females [68, 69]. Similarly, the expression of *cyp19a1b* showed significant sexual dimorphism before and after gonadal differentiation in red-tail catfish. A role for *cyp19a1b* in gonadal differentiation has been revealed in north African catfish [70]. As for *hsd17b1,* a key enzyme in steroid hormone biosynthesis, may play an essential role in estrogen synthesis in the ovary [71–76]. The expression of *cyp19a1* may be regulated by *foxl2* [43, 77], and the expression pattern of *hsd17b1* was similar to that of *cyp19a1b* in red-tail catfish. So *foxl2* may regulate the expression of both *hsd17b1* and *cyp19a1b*. *Cyp19a1* and *foxl2* were upregulated in sex-reversed females compared to wild-type males, suggesting that *cyp19a1* and *foxl2* were associated with ovarian development in yellow catfish (*Pelteobagrus fulvidraco*) [30]. *Foxl2* was involved in gonadal differentiation and the maintenance of ovarian function, and its expression was upregulated in the female in the early stage of ovarian differentiation [78, 79]. The expression of *foxl2* in red-tail catfish was consistent with the above findings in other fish. Knockdown of *foxl2* can lead to complete sexual reversal in zebrafish, gibel carp (*Carassius gibelio*) and Nile tilapia (*Oreochromis niloticus*) [25, 80–82]. Therefore, we speculate that *foxl2*, *cyp19a1b*, and *hsd17b1* may be female-biased genes and may be involved in the sex determination process in red-tail catfish.

Besides above candidate sex determination genes, *tsga10* was identified from DEGs. Originally isolated from adult testis, *tsga10* is over 80 kb in length and consists of 19 exons, and it may be involved in the maintenance of normal sperm structure and spermatogenesis [10, 83–86]. The expression of *tsga10* was up-regulated at four developmental stages, but the overall expression level of the gene was very low in both sexes. This result was similar to that found in zebrafish (*Danio rerio*) [87]. What’s more, *tsga10* knockout could cause infertility in male mice, resulting in disordered mitochondrial sheath formation and significantly reduced sperm motility [88]. There are no reports on *tsga10* in relation to sex determination or differentiation in fish. Therefore, whether *tsga10* is involved in sex determination or gonadal development in red-tail catfish needs to be further explored in future.

## Conclusions

RNA-seq was used to identify candidate genes involved in sex determination and to reveal their expression levels at four different stages of gonadal development. Seven DEGs shared by the four developmental stages were identified, of which *amh* and *gdf6a* may be the male-biased expression genes, while *foxl2*, *cyp19a1b* and *hsd17b1* may be the female-biased expression genes, which were involved in steroid hormone biosynthesis and TGF-β signaling pathways. Our results will provide insight into evolutionary processes of sex determination mechanisms in fish, as well as a useful genetic basis for sex-control breeding to produce monosexual populations.

## Methods

### Fish culture and ethics statement

Red-tail catfish were reared to be sexually mature in the Xishuangbanna Indigenous Fish Breeding Center and one full-sib family was established. After hatching there, fries were transferred to the National Aquatic Biological Resource Center (NABRC, Wuhan, China) and cultured in re-circulating aerated freshwater tanks at 26℃ prior to sample collection. Fries were fed three times daily with hatched artemia nauplii for the first two weeks, mixtures of artemia nauplii and frozen bloodworms for the next week, both frozen bloodworms and pellet feed for two weeks, then pellet feed for the following culture. All experiments and animal treatments were carried out according to the principles of Animal Care and Use Committee of Institute of Hydrobiology, Chinese Academy of Sciences.

### Gonadal histology and sample collection

All sampled individuals were firstly euthanized using an overdose of MS222 before gonad sample collection at different developmental stages of red-tail catfish. Gonads were fixed with paraformaldehyde (PFA) for more than 24 h at 4℃, then dehydrated, embedded in paraffin for section. After the slices were deparaffinized and rehydrated, they were stained with hematoxylin and eosin solutions. Microphotography was performed using a microscope from Carl Zeiss (Axio Imager M2). Significant stages of the early gonad development were determined based on histological analysis of gonads at different developmental stages. Sexual phenotypes of all sampled individuals were determined based on the sex-specific genetic markers of red-tail catfish [11], and the results of the sex identification were shown in Fig. S5 (with the samples of 48dph as an example). In this study, four developmental stages (10dph, 18dph, 30dph, and 48dph) were set up with a total of 24 samples. The first developmental stage (10dph) included the male gonadal tissue sample “M10dph” and the female gonadal tissue sample “F10dph”, and 27 gonads pooled into one sequencing sample for each sex. At the second developmental stage (18dph), the male gonadal tissue sample “M18dph” and the female gonadal tissue sample “F18dph” were sampled, and 15 gonads were pooled into one sample for each sex. The male gonadal tissue sample “M30dph” and the female gonadal tissue sample “F30dph” consisted of 12 testes and ovaries at the third developmental stage (30dph), respectively. At the fourth developmental stage (48dph), 12 testes and ovaries were pooled into the male gonadal tissue sample “M48dph” and the female gonadal tissue sample “F48dph”, respectively. Each of sequencing samples at different stages was performed with three replicates. Gonad samples were carefully collected and immediately placed in RNAprotect Tissue Reagent, kept at 4 ℃ overnight, and then store at -20 ℃ until RNA extraction.

### RNA extraction, library construction and sequencing

Total RNA was extracted using the mirVana miRNA Isolation Kit (Ambion, USA) following the manufacturer’s protocol. RNA purity and quantification were evaluated using the NanoDrop 2000 spectrophotometer (Thermo Scientific, USA). RNA integrity was evaluated using the Agilent 2100 Bioanalyzer (Agilent Technologies, USA). The samples with RNA Integrity Number (RIN) ≥ 7 were subjected to the subsequent analysis. The cDNA libraries were constructed using TruSeq Stranded mRNA LTSample Prep Kit (Illumina, USA) according to the manufacturer’s instructions. In brief, after the total RNA was extracted and digested by DNase, the mRNA was enriched by magnetic beads with Oligo (dT). Adding Fragmentation Buffer to break the mRNA into short fragments, which was used as template to synthesize first-strand cDNA with random hexamer primer. Then, a two-strand synthesis reaction system was prepared to synthesize second-strand cDNA, and second-strand cDNAs were purified by beads from kit. The purified double-stranded cDNA was then subjected to terminal repair, poly(A) addition and sequencing splicing, fragment size selection and PCR amplification. These RNA-Seq libraries were sequenced on the BGI DNBSEQ-T7 sequencing platform (Shanghai OE Biomedical Technology Company Limited, China) and 150 bp paired-end reads were generated.

### Gonad transcriptome assembly and annotation

Raw data (raw reads) of fastq format were firstly processed using Trimmomatic (v 0.36) [89] and the reads containing ploy-N and the low quality reads were removed to obtain the clean reads (LEADING:3 TRAILING:3 ILLUMINACLIP:TruSeq3-PE-2.fa:2:30:10:8:true SLIDINGWINDOW:4:15 MINLEN:50). Then clean reads were mapped to the reference genome of red-tail catfish (PRJNA842523 and PRJNA841381) using Hisat2 (v 2.2.1.0) [90]. The reads with a perfect match were further used for subsequent annotation analysis based on the reference genome.

### Differential expression analysis and DEGs identification

Relative gene expression levels of each gene were characterized by fragments per kilobase of transcript per million mapped reads (FPKM) [91]. FPKM of each gene was calculated and the read counts of each gene were obtained by HTSeq-count (v 0.9.1) [92]. Differential expression analysis between two sexes at four developmental stages was performed using the DESeq2 (v 1.20.0) (DESeqDataSetFromMatrix(countData = countData, colData = colData, design =  ~ condition)) [93, 94]. Genes with *p*-value < 0.05 and fold change > 2 or fold change < 0.5 were assigned as the threshold for DEGs. Gene ontology (GO) enrichment and Kyoto encyclopedia of genes and genomes (KEGG) pathway enrichment analysis of DEGs were performed to screen the significantly enriched term using R (v 3.2.0) based on the hypergeometric distribution [95–98]. In this study, the expression patterns of four developmental stages were revealed, and up-regulated/down-regulated was determined by comparing the male with the female. Unless otherwise stated, up-regulated refers to the expression in the male was higher than in the female, while down-regulated refers to the expression in the male was lower than in the female.

### RT-qPCR verification of expression patterns

Seven candidate DEGs were selected from the four developmental stages to verify the reliability of RNA-seq data by RT-qPCR. The primers for these genes (Table 3) were designed using Primer Premier (v 5.0) [99]. The PrimeScript™ RT reagent Kit with gDNA Eraser (TaKaRa) was used for cDNA synthesis following the manufacturer’s instructions. RT-qPCR was performed on a Step One real-time PCR System (Roche, USA) with iTaq Universal SYBR Green Supermix (BIO RAD). The β-actin gene was used as an endogenous reference gene and three biological replicates were performed for each reaction. The relative expression level was measured in terms of threshold cycle value and normalized using the 2^−∆∆Ct^ method [100]. For statistical analysis, SPSS (v 19.0) was used for significance test (*p* < 0.05).

**Table 3:** The sequences information of primers used for RT-qPCR

Gene ID	DEGs	Primer ID	Primer sequences (5′-3′)	Product size (bp)
Hw11G012730	*amh*	F	GGAGAAATACCTGCTGGAACC	171
		R	ATGTCGTCATACGCTATGGGC	
Hw15G016665	*gdf6a*	F	CCAACAGATGCCTGAAAGA	221
		R	TGACGATGATGACGACGAT	
Hw05G006197	*cyp17a*	F	CGTGGAGATCCTGAGTTTGA	283
		R	TCCTGGGTGCTTGTATTGTT	
Hw12G014257	*cyp19a1b*	F	CCTCCAAATTCCTATCAACG	175
		R	TGCTCCACAAGCCTCCCTAT	
Hw14G015812	*hsd17b1*	F	CATCAGATCCATCCAAAACA	297
		R	TGCGAATCGTACCCAGTAAA	
Hw21G022357	*foxl2*	F	GATTTTTAGTTTTCGGCTCGT	295
		R	AGGTCTGGTCTGGTGATTTTT	
Hw20G021635	*tsga10*	F	GAAGGAGTAAAGCAGGGTTTG	161
		R	GGGATGATTTTGGCAGTATGG	
Hw25G026100	*β-actin*	F	AGAGGTATCCTGACCCTGAAGTAC	328
		R	GAGCATAACCTTCATAGATGGGCACAG	

## Supplementary Information

**Additional file 1: Table S1.** The list of DEGs identified in M10dph-vs-F10dph.

**Additional file 2: Table S2.** The list of DEGs identified in M18dph-vs-F18dph.

**Additional file 3: Table S3.** The list of DEGs identified in M30dph-vs-F30dph.

**Additional file 4: Table S4.** The list of DEGs identified in M48dph-vs-F48dph.

**Additional file 5: Fig. S1.** The DEGs involved in the steroid hormone biosynthesis pathway in M10dph-vs-F10dph (ko00140, https://www.kegg.jp/pathway/map00140). The pink box in the figure represents up-regulated genes and the blue box represents down-regulated genes.

**Additional file 6: Fig. S2.** The DEGs involved in the steroid hormone biosynthesis pathway in M18dph-vs-F18dph (ko00140, https://www.kegg.jp/pathway/map00140). The pink box in the figure represents up-regulated genes and the blue box represents down-regulated genes.

**Additional file 7: Fig. S3. **The DEGs involved in the steroid hormone biosynthesis pathway in M30dph-vs-F30dph (ko00140, https://www.kegg.jp/pathway/map00140). The pink box in the figure represents up-regulated genes, the blue box represents down-regulated genes, and the olive box indicate that both up- and down-regulated genes are included.

**Additional file 8: Fig. S4.** The DEGs involved in the steroid hormone biosynthesis pathway in M48dph-vs-F48dph (ko00140, https://www.kegg.jp/pathway/map00140). The pink box in the figure represents up-regulated genes, the blue box represents down-regulated genes, and the olive box indicate that both up- and down-regulated genes are included.

**Additional file 9: Table S5. **The real *p*-values of RT-qPCR.

**Additional file 10: Fig. S5. **The electrophoretic pattern of the sex identification in 36 males and 36 females. (a): 351 bp Y‐specific fragment amplified by the Y‐specific primer pair 18‐Fy and 18‐Ry only in male individuals; (b): 727 bp Y‐specific fragment amplified by Y‐specific primer pair 20‐Fy and 20‐Ry only in male individuals. M1-M12: sample “M48dph1”, M13-M24: sample “M48dph2”, M25-M36: sample “M48dph3”. F1-F12: sample “F48dph1”, F13-F24: sample “F48dph2”, F25-F36: sample “F48dph3”. M: DL 2000 DNA marker.

## Data Availability

The data that support the results of this present study are available from the corresponding author upon reasonable request. The reference genome of red-tail catfish is available in the NCBI SRA database under the BioProject accession number PRJNA842523 (https://www.ncbi.nlm.nih.gov/sra/?term=PRJNA842523) and PRJNA841381 (https://www.ncbi.nlm.nih.gov/sra/?term=PRJNA841381).

## Abbreviations

*amh*: Anti-Mullerian hormone
*cyp17a*: Cytochrome P450 family 17 subfamily A
*cyp19a1b*: Cytochrome P450 family 19 subfamily A polypeptide 1b
CAMs: Cell adhesion molecules
dph: Days post hatching
DEGs: Differentially expressed genes
ESD: Environmental sex determination
F_dph: Female gonad samples at _ days post hatching
*foxl2*: Forkhead box L2
FPKM: Fragments per kilobase of transcript per million mapped reads
*gdf6a*: Growth differentiation factor 6a
GSD: Genotypic sex determination
GO: Gene ontology
*hsd17b1*: Hydroxysteroid 17-beta dehydrogenase 1
KEGG: Kyoto encyclopedia of genes and genomes
M_dph: Male gonad samples at _ days post hatching
PFA: Paraformaldehyde
RIN: RNA integrity number
RT-qPCR: Real-time quantitative PCR
*tsga10*: Testis-specific gene antigen 10
