# Supplementary figures and images for: Gonadal transcriptomes reveal sex-biased expression genes associated with sex determination and differentiation in red-tail catfish (Hemibagrus wyckioides)
Source: BMC Genomics. 2023 Apr 6;24:183. doi: 10.1186/s12864-023-09264-x (PMC10077648; doi:10.1186/s12864-023-09264-x)

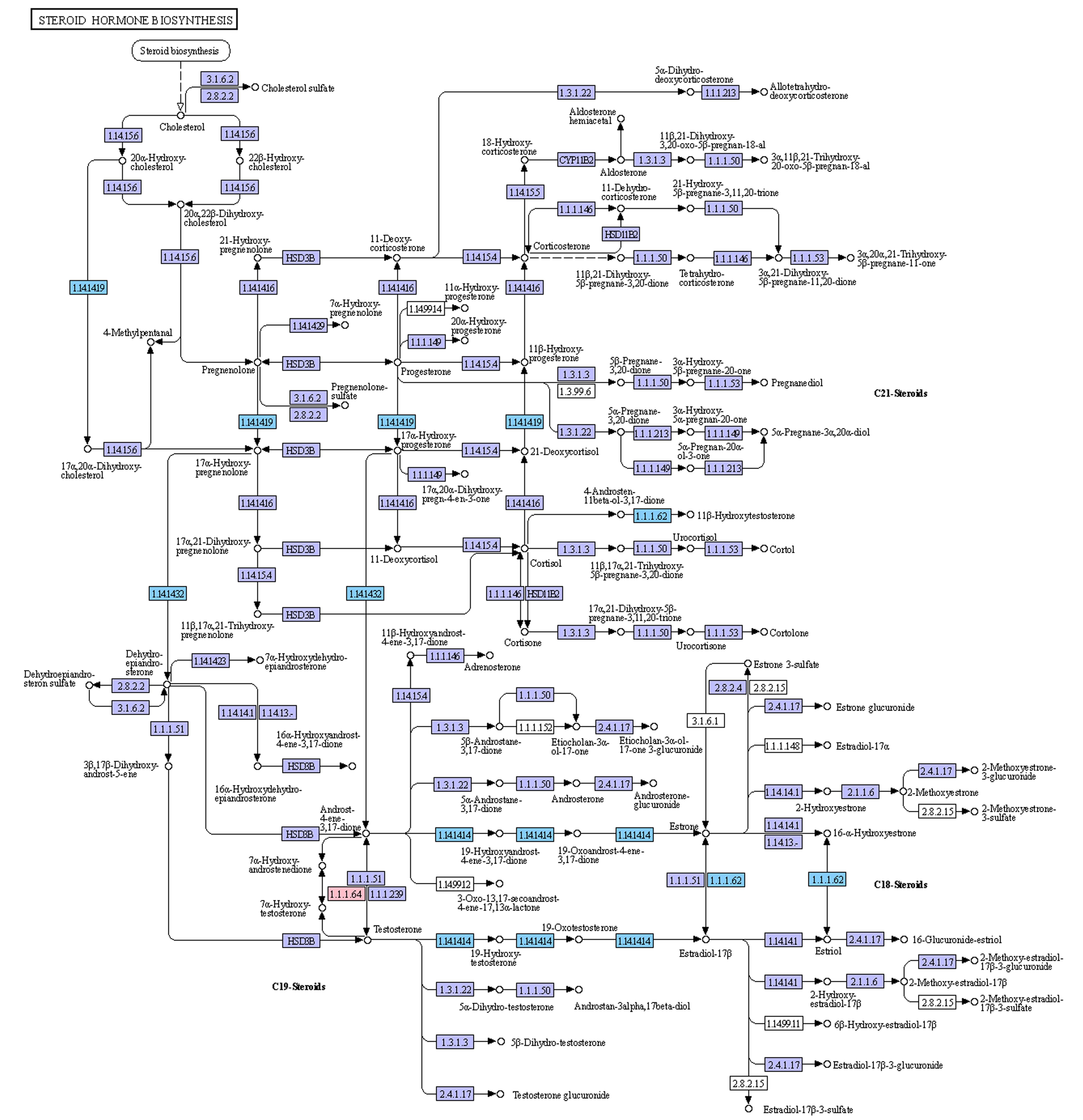

Supplement: Supplementary file 5 — Additional file 5: Fig. S1. The DEGs involved in the steroid hormone biosynthesis pathway in M10dph-vs-F10dph (ko00140, https://www.kegg.jp/pathway/map00140). The pink box in the figure represents up-regulated genes and the blue box represents down-regulated genes. [file 12864_2023_9264_MOESM5_ESM.tif]

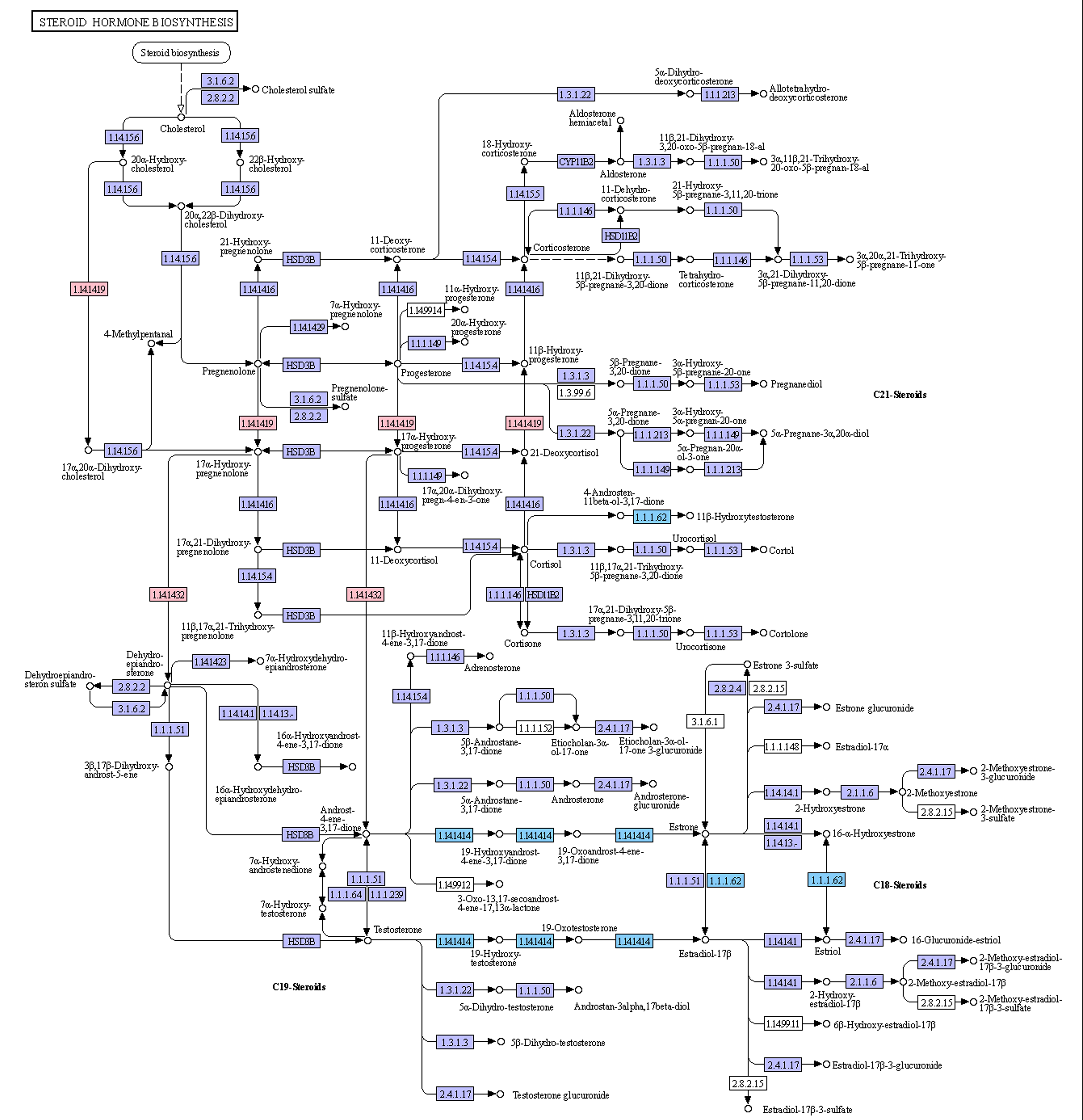

Supplement: Supplementary file 6 — Additional file 6: Fig. S2. The DEGs involved in the steroid hormone biosynthesis pathway in M18dph-vs-F18dph (ko00140, https://www.kegg.jp/pathway/map00140). The pink box in the figure represents up-regulated genes and the blue box represents down-regulated genes. [file 12864_2023_9264_MOESM6_ESM.tif]

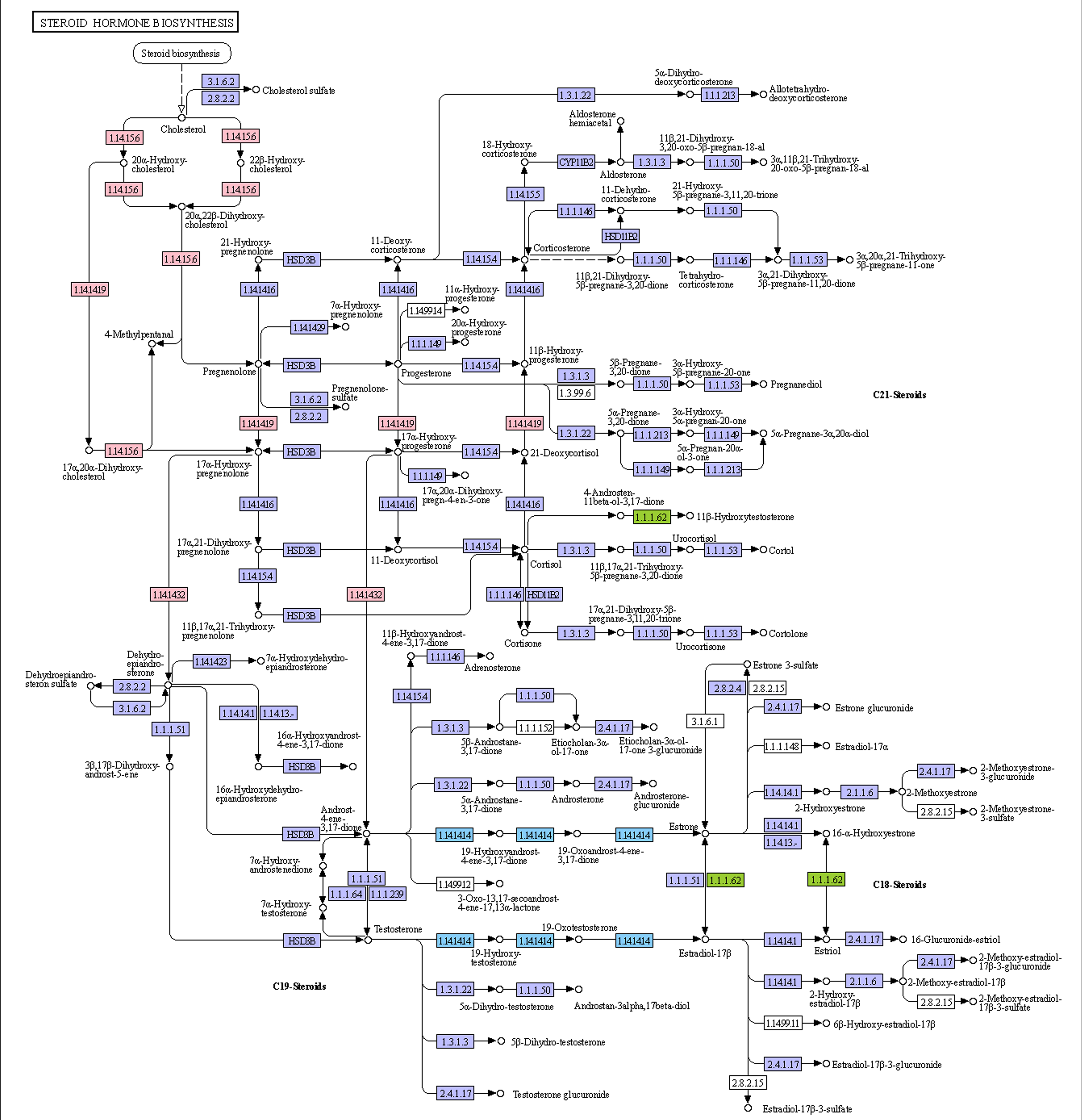

Supplement: Supplementary file 7 — Additional file 7: Fig. S3. The DEGs involved in the steroid hormone biosynthesis pathway in M30dph-vs-F30dph (ko00140, https://www.kegg.jp/pathway/map00140). The pink box in the figure represents up-regulated genes, the blue box represents down-regulated genes, and the olive box indicate that both up- and down-regulated genes are included. [file 12864_2023_9264_MOESM7_ESM.tif]

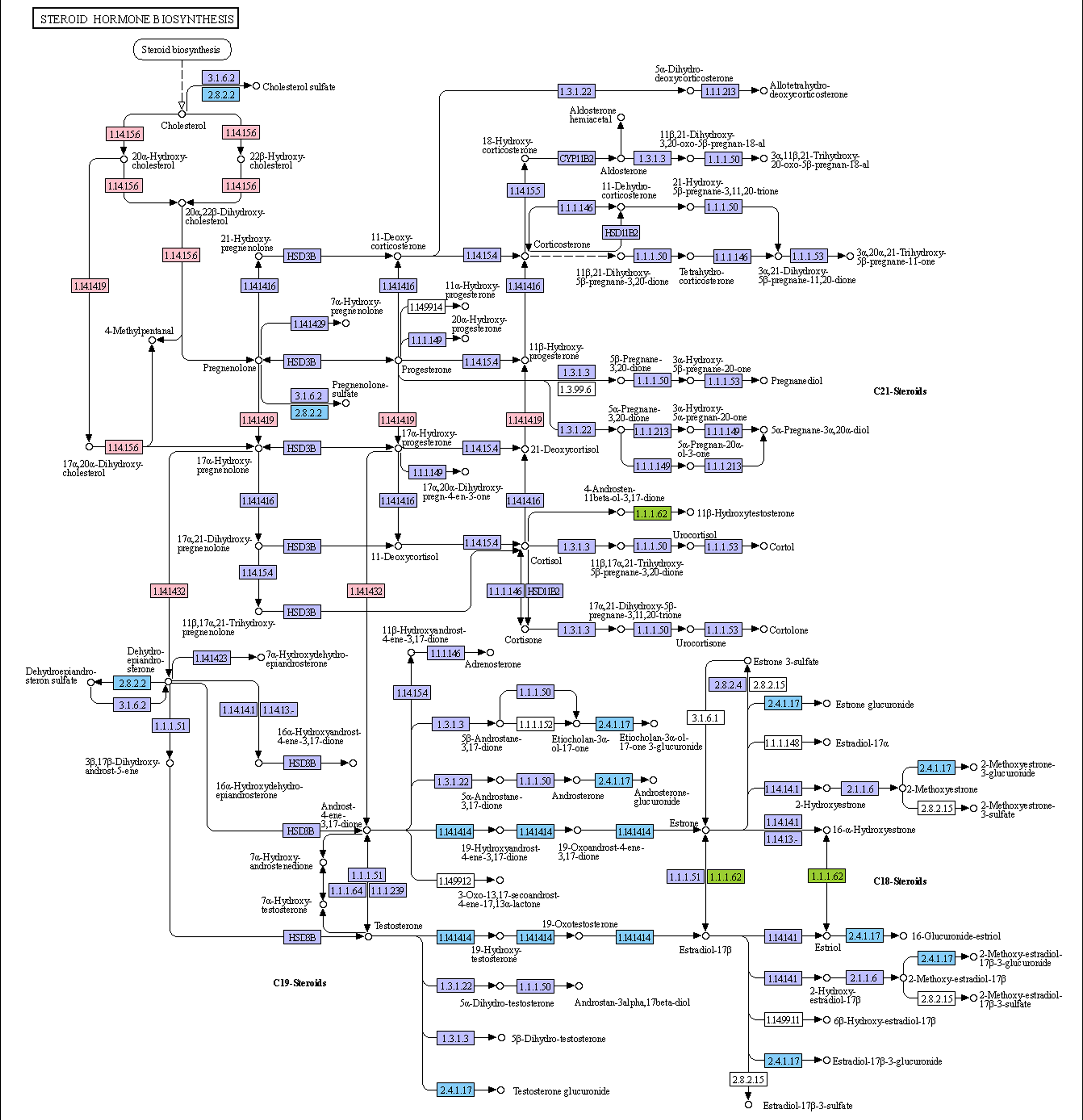

Supplement: Supplementary file 8 — Additional file 8: Fig. S4. The DEGs involved in the steroid hormone biosynthesis pathway in M48dph-vs-F48dph (ko00140, https://www.kegg.jp/pathway/map00140). The pink box in the figure represents up-regulated genes, the blue box represents down-regulated genes, and the olive box indicate that both up- and down-regulated genes are included. [file 12864_2023_9264_MOESM8_ESM.tif]

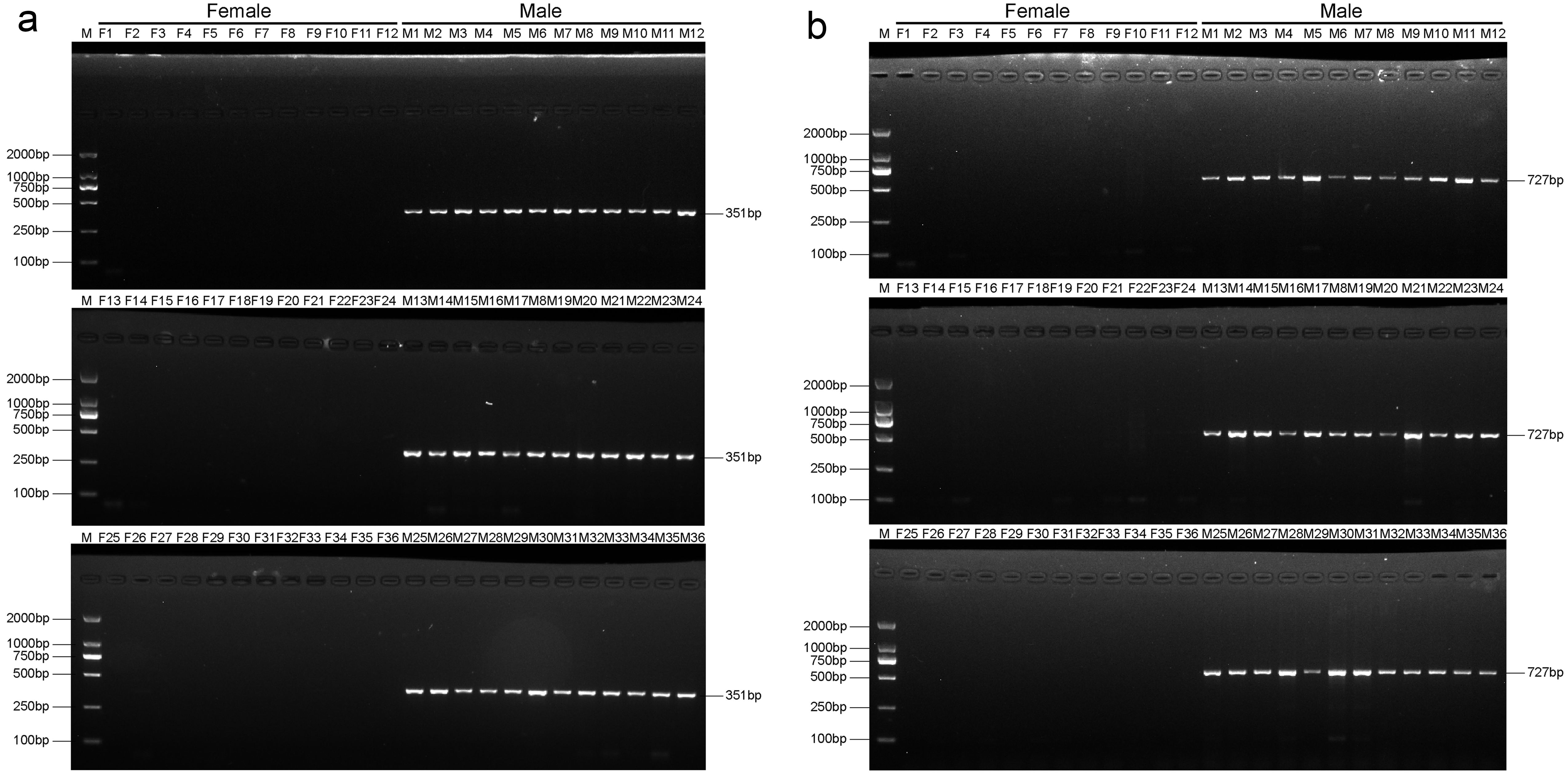

Supplement: Supplementary file 10 — Additional file 10: Fig. S5. The electrophoretic pattern of the sex identification in 36 males and 36 females. (a): 351 bp Y‐specific fragment amplified by the Y‐specific primer pair 18‐Fy and 18‐Ry only in male individuals; (b): 727 bp Y‐specific fragment amplified by Y‐specific primer pair 20‐Fy and 20‐Ry only in male individuals. M1-M12: sample “M48dph1”, M13-M24: sample “M48dph2”, M25-M36: sample “M48dph3”. F1-F12: sample “F48dph1”, F13-F24: sample “F48dph2”, F25-F36: sample “F48dph3”. M: DL 2000 DNA marker. [file 12864_2023_9264_MOESM10_ESM.tif]
